# Phenylalanine ammonia-lyase2.1 contributes to the soybean response towards *Phytophthora sojae* infection

**DOI:** 10.1038/s41598-017-07832-2

**Published:** 2017-08-03

**Authors:** Chuanzhong Zhang, Xin Wang, Feng Zhang, Lidong Dong, Junjiang Wu, Qun Cheng, Dongyue Qi, Xiaofei Yan, Liangyu Jiang, Sujie Fan, Ninghui Li, Dongmei Li, Pengfei Xu, Shuzhen Zhang

**Affiliations:** 1 Soybean Research Institute, Key Laboratory of Soybean Biology of Chinese Education Ministry, Northeast Agricultural University, Harbin, Heilongjiang China; 2 Heilongjiang Academy of Land Reclamation Sciences, Harbin, Heilongjiang China; 3 First Affiliated Hospital of Harbin Medical University, Harbin, Heilongjiang China; 4 Soybean Research Institute of Heilongjiang Academy of Agricultural Sciences, Key Laboratory of Soybean Cultivation of Ministry of Agriculture P. R. China, Harbin, Heilongjiang China; 5 Jiamusi Branch Academy of Heilongjiang Academy of Agricultural Sciences, Jiamusi, Heilongjiang China

## Abstract

Phytophthora root and stem rot of soybean [*Glycine max* (L.) Merr.] caused by *Phytophthora sojae* is a destructive disease worldwide. Phenylalanine ammonia-lyase (PAL) is one of the most extensively studied enzymes related to plant responses to biotic and abiotic stresses. However, the molecular mechanism of PAL in soybean in response to *P*. *sojae* is largely unclear. Here, we characterize a novel member of the soybean PAL gene family, *GmPAL2*.*1*, which is significantly induced by *P*. *sojae*. Overexpression and RNA interference analysis demonstrates that GmPAL2.1 enhances resistance to *P*. *sojae* in transgenic soybean plants. In addition, the PAL activity in *GmPAL2*.*1*-OX transgenic soybean is significantly higher than that of non-transgenic plants after infection with *P*. *sojae*, while that in *GmPAL2*.*1*-RNAi soybean plants is lower. Further analyses show that the daidzein, genistein and salicylic acid (SA) levels and the relative content of glyceollins are markedly increased in *GmPAL2*.*1*-OX transgenic soybean. Taken together, these results suggest the important role of GmPAL2.1 functioning as a positive regulator in the soybean response to *P*. *sojae* infection, possibly by enhancing the content of glyceollins, daidzein, genistein and SA.

## Introduction

Plants have evolved multiple defense signaling pathways to respond to environment stress and pathogen attack^1^. The phenylpropanoid pathway is one of the important secondary metabolism pathways and produces a variety of secondary metabolites^2–6^. Phenylalanine ammonia-lyase (PAL) catalyzes the first step in the phenylpropanoid pathway^7^, in which L-phenylalanine undergoes deamination to yield trans-cinnamate and ammonia^8^. Formation of lignin, suberin, phytoalexins, stilbenes, coumarins and other flavonoids all depends on PAL activity^3, 9^. It has been reported that the biosynthesis of glyceollins occurs via the phenylpropanoid pathway in soybean^7^. Glyceollins are soybean-derived phytoalexins that accumulate in the seeds in response to environmental stimulus^10^. In general, glyceollins protect plant tissues from environmental challenges, possibly by reducing the oxidative damage induced by stress factors. Thus, these compounds have significant cellular anti-oxidative activities^10–12^. Moreover, glyceollins also inhibit several pathogen species and the growth of *Phytophthora megasperma* var. *sojae* ^13–15^. Research has revealed that glyceollins are a major factor in the restriction of *Phytophthora sojae* during compatible and incompatible interactions of soybean with the pathogen^16^. The accumulation of glyceollins is also related to deploying the defense responses to the cell wall glucan elicitor from the pathogen *P*. *sojae* in soybean^17, 18^. There are several enzymes involved in the glyceollin biosynthetic pathways^19^. PAL acts as a key-control enzyme, which catalyzes the first step in the synthesis of glyceollins^20–23^.

PAL is present in all higher plants studied and has also been found in some fungi^24, 25^ and cyanobacteria^26^. However, it has not yet been detected in *Eubacteria*, *Archaea*, and animals^27, 28^. In plants, PAL is encoded by a multi-gene family. Four PAL isoforms have been detected in Arabidopsis^29, 30^, two in Rubus^31^, five in Populus^32^, seven in cucumber^33^, and twelve in watermelon^34^. Other research has shown that the co-expression of different tobacco PAL proteins in *Escherichia coli* can produce functional heterotetrameric enzymes^35^. In soybean, PAL is encoded by a small gene family ranging from 2 to 3 members and can be divided into different subgroups^36^.

However, homo- or heterotetramers of the PAL protein and the different *PAL* genes are thought to be involved in plant development and in the response to different stress stimuli^37^. For instance, RiPAL1 is associated with early fruit ripening and the biosynthesis of flavonoids, whereas RiPAL2 correlates with late stages of flower and fruit development and the accumulation of anthocyanins in Rubus^31^. *PtPAL1* is expressed in non-lignified tissues of shoots and roots, whereas *PtPAL2* is expressed in the heavily lignified structural cells of shoots and in non-lignified cells of root tips in aspen^38^. Alternatively, several studies indicate that the gene expression of *PAL* is stimulated during developmental programming and by a variety of environmental stresses, including pathogenic attacks, wounding, UV irradiation, low temperatures, and low levels of nitrogen, phosphate, or ions^3, 39–42^. In French bean, an expression study of *PvPAL* showed that *PvPAL* is induced with tissue wounding and activated by fungi attack, suggesting that PAL may play a role in body injury and fungal responses^43^. In Arabidopsis, *PAL1* and *PAL2* double mutants are more sensitive to ultraviolet-B light and show a significant reduction in lignin accumulation than wild type plants^44^. In rice, a recent report shows that *OsPAL4* and possibly *OsPAL6* are key contributors to a broad spectrum of disease resistance^45^. Although PAL is extensively studied in various plants, the systematical research on PAL2 in soybean disease-resistance has not been reported.

In a previous study, a cDNA library enriched for mRNAs encoding ESTs that were increased in abundance during infection with *P*. *sojae* was constructed by suppression subtractive hybridization from leaf tissues of the highly resistant soybean cultivar ‘Suinong 10’, and an EST homologous to a phenylalanine ammonia-lyase from *Lotus japonicus* was identified to be upregulated by microarray and real-time PCR^46^. In the present work, the full-length EST, designated *GmPAL2*.*1* (GenBank accession no. NM_001250027, NCBI protein no. NP_001236956), was isolated using RT-PCR from ‘Suinong 10’ soybean. The expression patterns of *GmPAL2*.*1* induced under abiotic and biotic stresses were examined. To gain insight into the function of GmPAL2.1 in soybean, the *GmPAL2*.*1* gene was overexpressed in soybean plants under the control of the35S promoter, and RNA interference (RNAi) technology was applied to suppress the expression of *GmPAL2*.*1* to generate knockdown soybean plants. Furthermore, the contents of SA and three kinds of isoflavone—daidzein, glycitein, and genistein—were analyzed. The relative content of glyceollins and the PAL activity in the transgenic plants were also investigated. Taking our findings together, we report insights into the function of a PAL gene in soybean, *GmPAL2*.*1*, in the defense response against *P*. *sojae*.

## Results

### Isolation and sequence analysis of *GmPAL2*.*1*

The full-length cDNA sequence of the *GmPAL2*.*1* gene (GenBank accession no. NM_001250027) was isolated from the total RNA of ‘Suinong 10’ using RT-PCR. Sequence analysis suggests that the full length of *GmPAL2*.*1* is 2284 bp and contains an open reading frame (ORF) encoding a polypeptide of 717 amino acids (Supplementary Fig. 1), with a predicted molecular mass of 78.116 kDa and a theoretic isoelectric point (pI) of 5.83. Phylogenetic tree and alignment analyses revealed that GmPAL2.1 has 71–87% identity for its overall amino acid sequence to *Lotus japonicus* LjPAL (BAF36972.1), *Astragalus membranaceus* AmPAL (EF567076.1), *Medicago truncatula* MtPAL (XM_003590423.1), *Trifolium pratense* TpPAL (AB236800.1), *Manihot esculenta* MePAL (AAK62030.1), *Daucus carota* DcPAL (BAG31931.1), *Fragaria vesca* FvPAL (XM_004304392.1), *Morus alba* MaPAL (HM064433.1), *Robinia pseudoacacia* RpPAL (ACF94716.1), *Pisum sativum* PsPAL (D10001.1), *Oryza sativa* OsPAL (NM_001054017.1) and *Arabidopsis thaliana* AtPAL (NM_111869.3) (Supplementary Fig. 2A,C). In addition, there are eight *GmPAL* members identified in soybean genomes^47, 48^. *GmPAL2*.*1* shares 97.07%, 88.56%, 88.15%, 87.64%, 83.15%, 83.01% and 73.41% identity with *GmPAL2*.*3* (XM_003542493), *GmPAL1*.*1* (XM_003554334), *GmPAL1*.*2* (XM_003521349), *GmPAL1*.*3* (XM_003521348), *GmPAL2*.*2* (XM_003556190), *GmPAL2*.*4* (XM_006589357) and *GmPAL3*.*1* (XM_003518532) at the amino acid level, respectively (Supplementary Fig. 3). The prediction of the three-dimensional (3D) structure of GmPAL2.1 based on the data from Phyre (http://www.sbg.bio.jc.ac.uk/phyre/) shows that this protein is a HAL/PAL-like family member that belongs to the L-aspartase-like superfamily (Supplementary Fig. 2B).

### Transcript abundance of *GmPAL2*.*1* under various stresses

**Figure 1:**
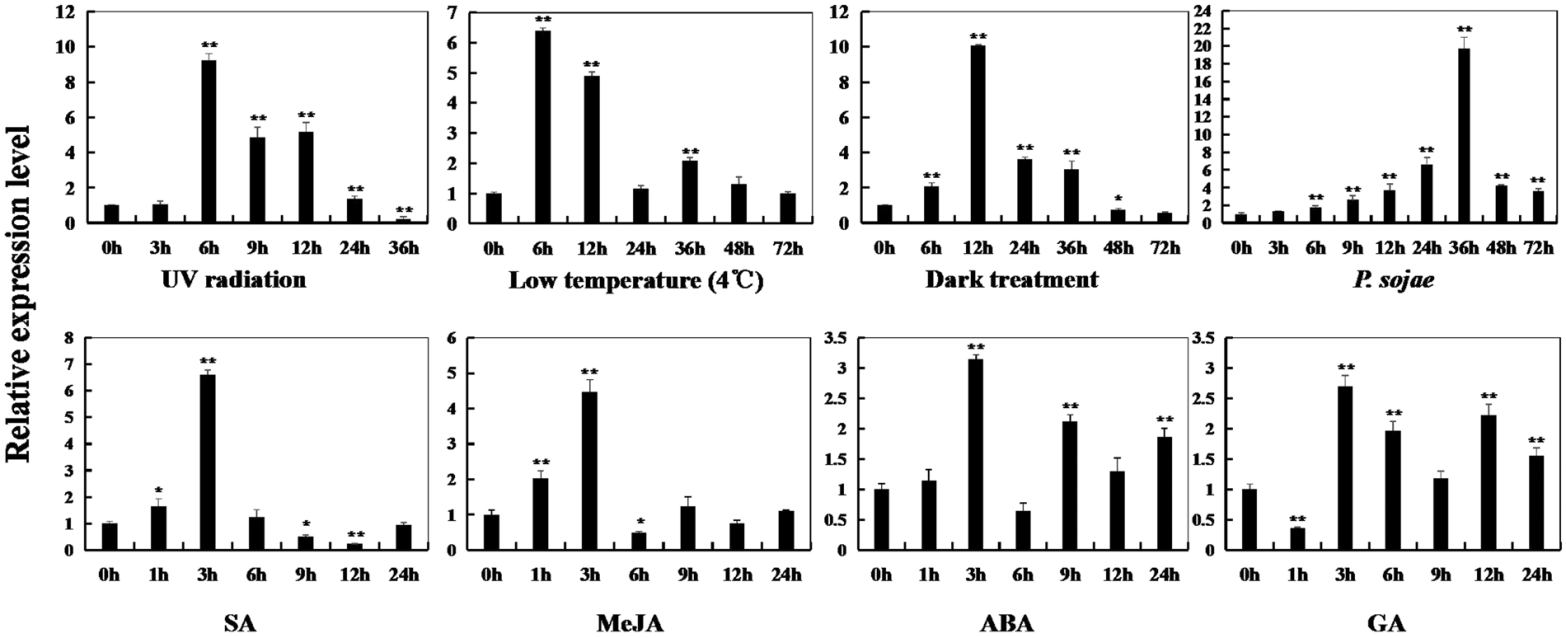
The relative transcript levels of *GmPAL2*.*1* at various time points post-treatment with SA, MeJA, ABA, GA, UV radiation, low temperature (4 °C), darkness and *P*. *sojae* ‘Suinong 10’ soybean plants. Fourteen-day-old plants were used for the treatments and analyses. The amplification of the soybean *Actin* (*GmActin4*) gene was used as an internal control to normalize all the data. The relative transcript levels of *GmPAL2*.*1* were quantified compared with mock plants at the same time points. The experiment was performed on three biological replicates with their respective three technical replicates and statistically analyzed using Student’s t-test (*P < 0.05; **P < 0.01). Bars indicate the standard error of the mean.

To evaluate the expression pattern of *GmPAL2*.*1*, quantitative RT-PCR was used to examine the transcript abundance of *GmPAL2*.*1* in ‘Suinong 10’ plants (resistant cultivar) and ‘Dongnong 50’ plants (susceptible cultivar). In ‘Suinong 10’ plants, quantitative real-time PCR shows that *GmPAL2*.*1* is induced by treatment with SA, MeJA, ABA and GA (Fig. 1). Under UV radiation, a low-temperature treatment (4 °C) and dark treatments, the transcripts of *GmPAL2*.*1* mRNA increase and reach a maximum level at 6, 6 and 12 h, respectively (Fig. 1).

**Figure 2:**
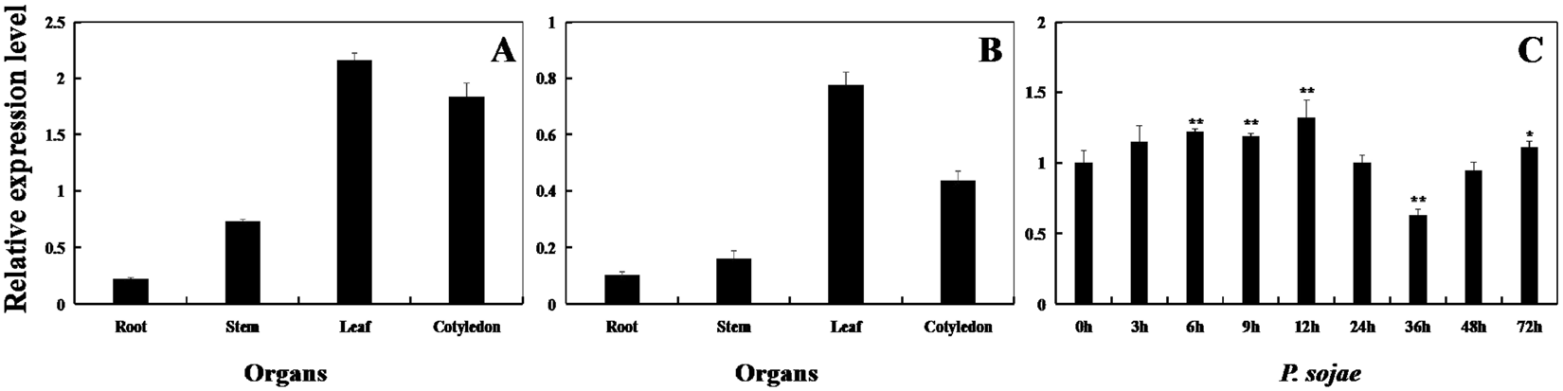
Expression patterns of *GmPAL2*.*1* in ‘Dongnong 50′ soybean plants. (**A**) The transcript abundance of *GmPAL2*.*1* in various tissues of ‘Suinong 10’ soybean under normal condition. (**B**) The transcript abundance of *GmPAL2*.*1* in various tissues of ‘Dongnong 50’ soybean under normal conditions. (**C**) The transcript levels of *GmPAL2*.*1* in ‘Dongnong 50’ soybean under *P*. *sojae* treatment. The roots, stems, leaves and cotyledons were prepared from 14-day-old seedlings. The relative transcript levels of *GmPAL2*.*1* were quantified compared with mock plants at the same time points. The amplification of the soybean *Actin* (*GmActin4*) gene was used as an internal control to normalize the data. For each sample, three biological replicates were analyzed with their respective three technical replicates. Bars indicate the standard error of the mean.

The tissue-specific transcript abundance of *GmPAL2*.*1* in ‘Suinong 10’ and ‘Dongnong 50’ shows that *GmPAL2*.*1* is constitutively and highly expressed in the leaves followed by the cotyledons, stems, and roots (Fig. 2A,B). The transcript levels of *GmPAL2*.*1* were also determined after treatment with *P*. *sojae*. A significant upregulation of *GmPAL2*.*1* expression is detected in the leaves at 6 h after the treatments and reaches a maximum level at 36 h, followed by a rapid decline in ‘Suinong 10’ (Fig. 1). However, it is slightly down-regulated at 36 h in ‘Dongnong 50’ plants, revealing differential expression for *GmPAL2*.*1* in resistant and susceptible cultivars (Fig. 2C).

### Subcellular localization of the GmPAL2.1 protein

**Figure 3:**
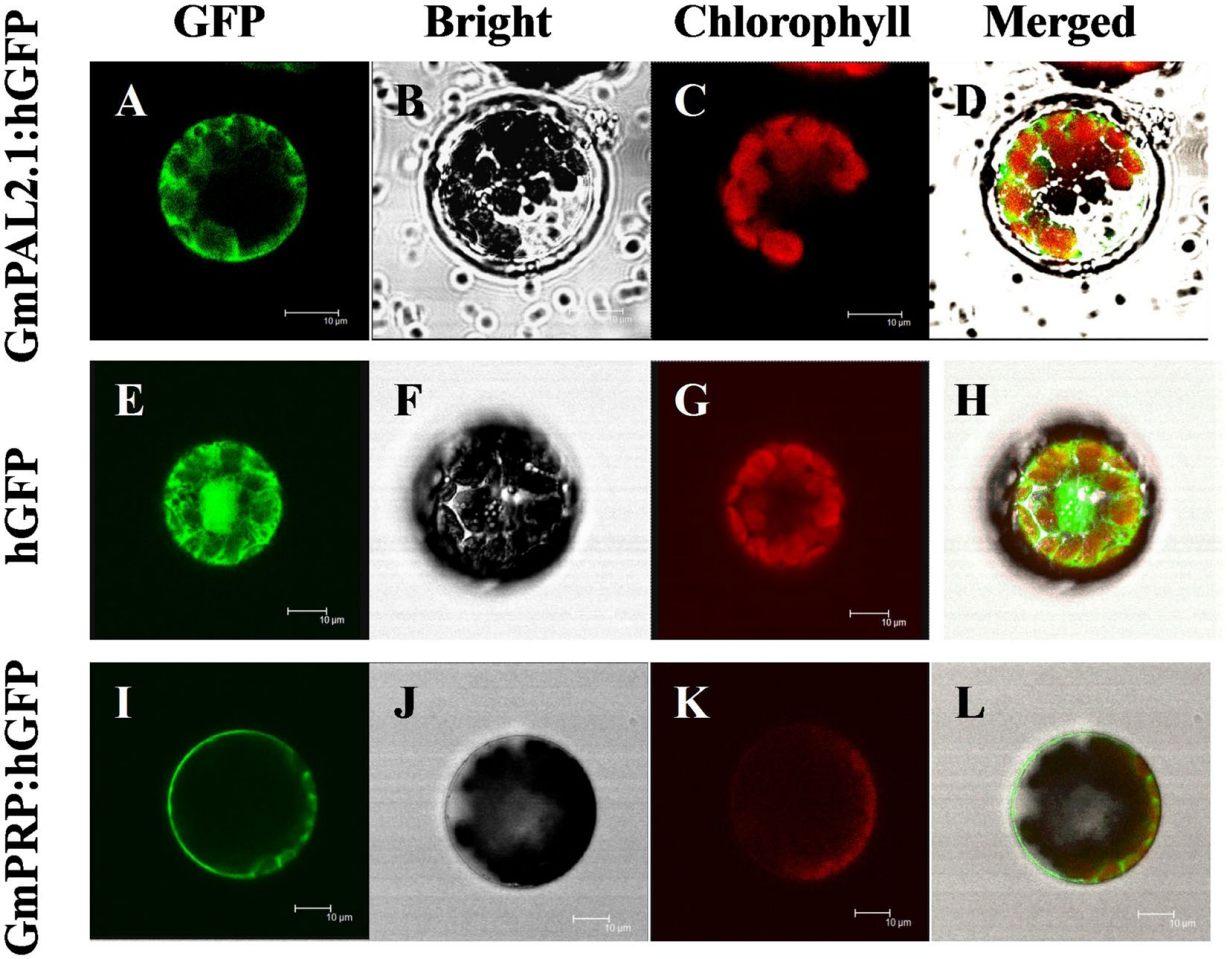
Subcellular localization analysis of the GmPAL2.1-GFP protein in Arabidopsis protoplasts. Subcellular localization was investigated in Arabidopsis protoplasts using a confocal microscope. The images of bright-field (**B**,**F** and **J**), the GFP fluorescence (green) only (**A**,**E** and **I**), the chlorophyll autofluorescence (red) only (**C**,**G** and **K**) and combined ones (**D**,**H** and **L**) are shown. All scale bars indicate 10 µm.

To determine the subcellular localization of the GmPAL2.1 protein, Arabidopsis protoplasts were examined to analyze the expression of GmPAL2.1-GFP fusion protein by the control of the 35S promoter. As shown in Fig. 3, confocal laser scanning microscopy reveals that GFP fluorescence is dispersed throughout the entire cell that was bombarded with the control plasmid 35S. GFP and the fusion GmPAL2.1-GFP protein are observed in the cell membrane and cytoplasm, similar to GmPRP^49^, indicating that GmPAL2.1 is present in both the cell membrane and cytoplasm. It should be noted that PAL was mainly located in cytoplasm and chloroplast, mitochondria, glyoxysome, peroxisoame, and other membrane organelles^50^. However, the enzymes encoded by different *PAL* genes could differ in subcellular location, and membrane associated PAL might’channel’cinnamic acid through interactions with membrane protein cinnamate 4-hydroxylase (C4H) for the second step in phenylpropanoid biosynthesis^51, 52^.

### Resistance to *P*. *sojae* in transgenic soybean plants

**Figure 4:**
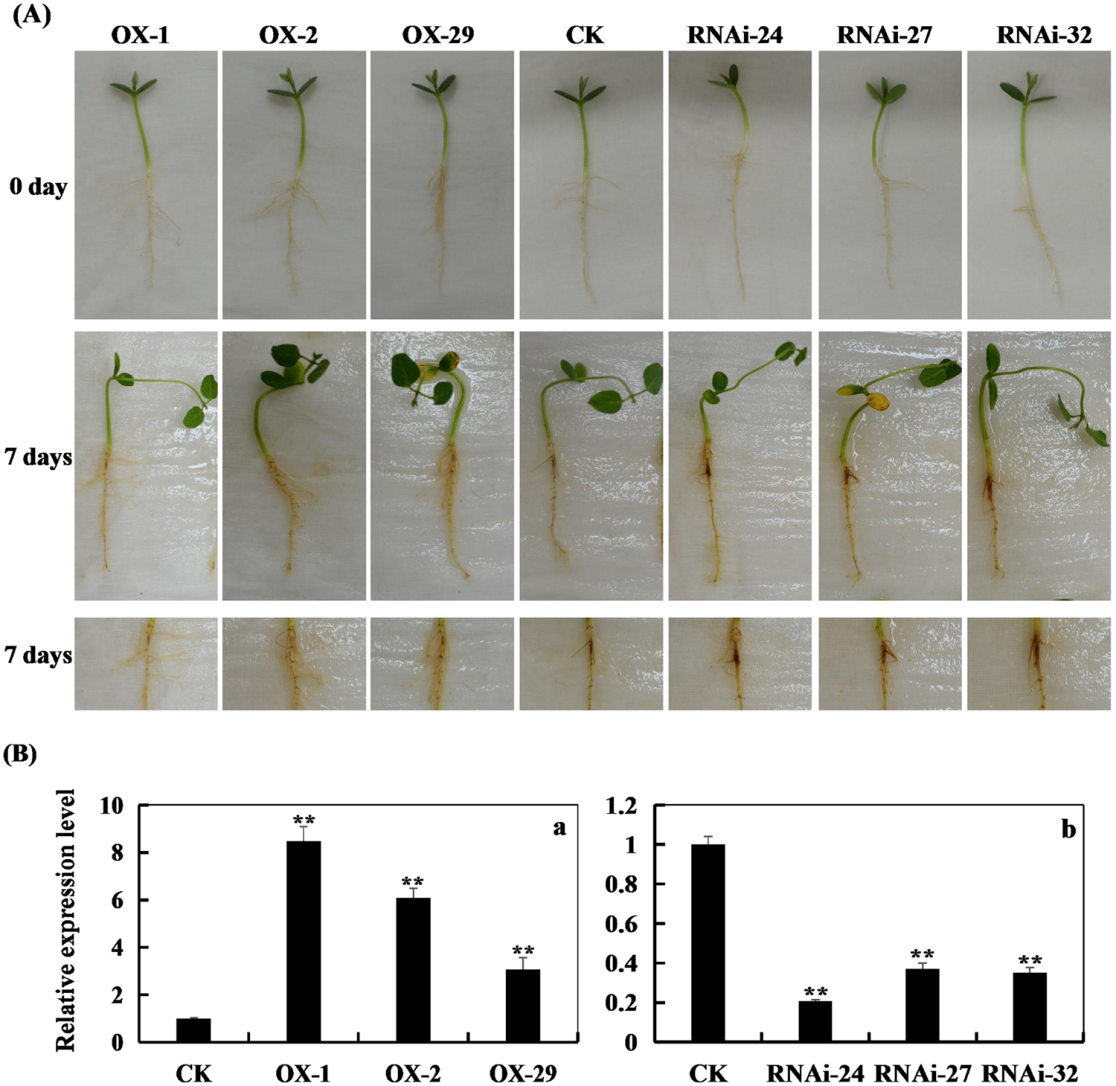
GmPAL2.1 enhances resistance to *P*. *sojae* in transgenic soybean roots. (**A**) Disease symptoms on the roots of the transgenic lines and non-transgenic lines treated with *P*. *sojae* at 7days. (**B**) qRT-PCR was used to determine the relative abundance of *GmPAL2*.*1* in three *GmPAL2*.*1*-overexpressing soybean plants (a) and three *GmPAL2*.*1*-RNAi soybean plants (b) Non-transgenic soybean plants were used as controls. For each sample, three biological replicates were analyzed with their respective three technical replicates and statistically analyzed using Student’s t-test (*P < 0.05, **P < 0.01). Bars indicate the standard error of the mean.

**Figure 5:**
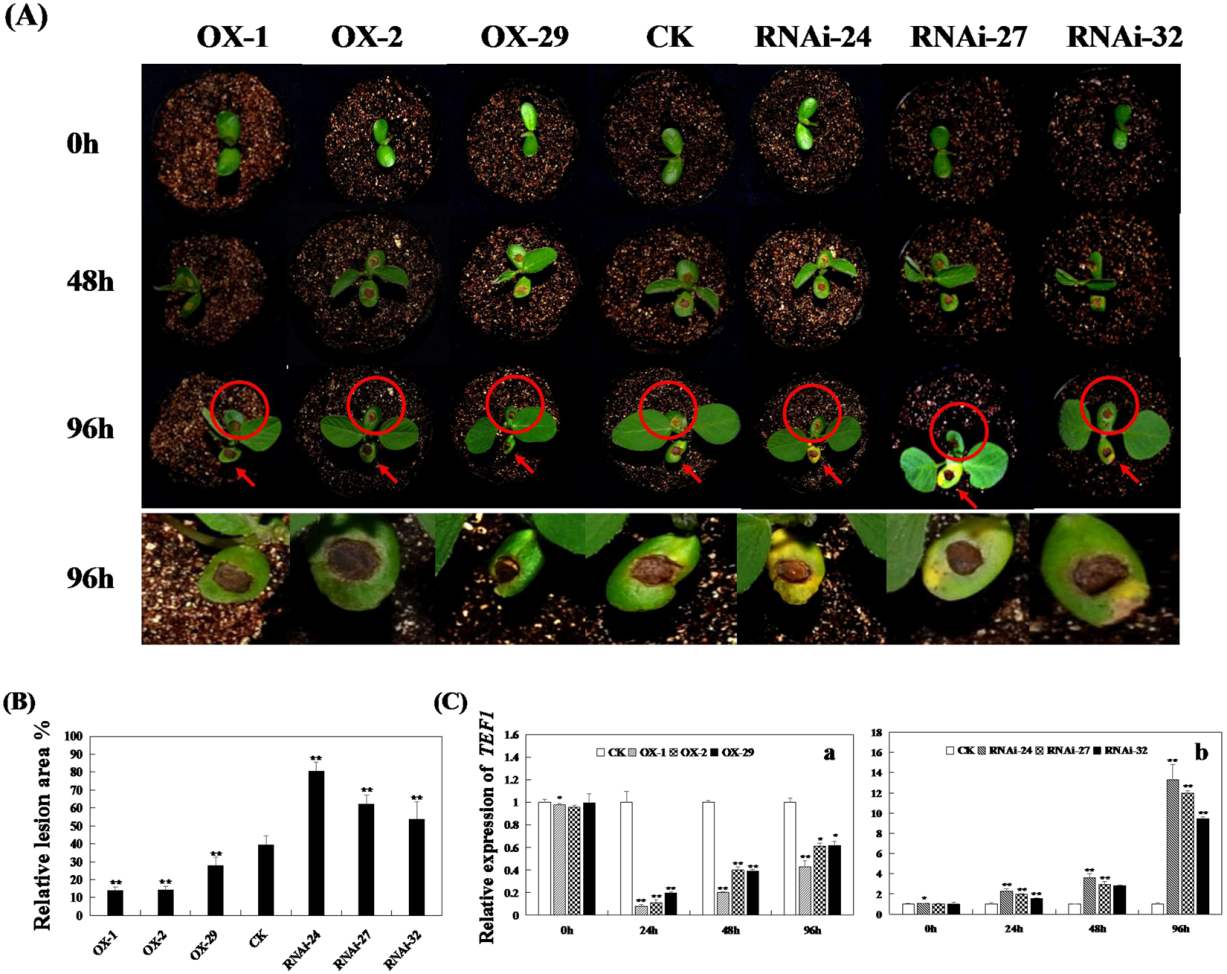
GmPAL2.1 enhances resistance to *P*. *sojae* in transgenic soybean cotyledons. (**A**) Disease symptoms on the living cotyledons of transgenic lines and non-transgenic lines treated with *P*. *sojae* inoculum at 48 h and 96 h. (**B**) The relative lesion area of transgenic soybean cotyledon infection with *P*. *sojae* after 96 h. The average lesion area of each independent transgenic line (n = 3) was calculated, and their relative lesion areas are shown in columns after a comparison with the average lesion area on non-transgenic soybean. (**C**) Quantitative real-time PCR analysis of the *P*. *sojae* relative biomass in three GmPAL2.1-overexpressing soybean plants (a) and three GmPAL2.1-RNAi soybean plants (b) based on the transcript level of the *P*. *sojae TEF1* gene. The experiment was performed on three biological replicates with their respective three technical replicates and statistically analyzed using Student’s t-test (*P < 0.05, **P < 0.01). Bars indicate the standard error of the mean.

To investigate whether the relative expression of *GmPAL2*.*1* in soybean has an effect on Phytophthora root rot resistance, six T_2_ transgenic soybean plants (namely, OX-1, OX-2, OX-29, RNAi-24, RNAi-27 and RNAi-32), of which the T_1_ transgenic soybean plants was confirmed through PCR and Southern hybridization (Supplementary Fig. 4), were selected. Real-time PCR was used to assay the pathogen response (Fig. 4B). As shown in Fig. 4A, six transgenic soybean plants showed that GmPAL2.1 enhances resistance to *P*. *sojae* after root infection. As shown in Fig. 5A, after 96 h of incubation with *P*. *sojae*, the cotyledons of the *GmPAL2*.*1*-RNAi soybean plants exhibit clear and large lesions, and the lesion area of the *GmPAL2*.*1*-OX soybean lines is obviously milder than that of non-transgenic and *GmPAL2*.*1*-RNAi soybean lines. The lesion area of the six transgenic lines were different than that of non-transgenic soybean plants at 96 h after inoculation (Fig. 5B). Moreover, the relative biomass of *P*. *sojae* in infected cotyledons after 24 h, 48 h and 96 h of incubation with zoospore suspensions of *P*. *sojae* was also analyzed by qPCR. The results indicate that the biomass of *P*. *sojae*, based on the transcript level of the *P*. *sojae TEF1* gene, is significantly lower (P < 0.01) in the *GmPAL2*.*1*-OX soybean plants than that in non-transgenic and *GmPAL2*.*1*-RNAi soybean lines during the course of the infection (Fig. 5C). These results indicate that the expression of *GmPAL2*.*1* in soybean plants plays an important role in resistance to *P*. *sojae*.

### Analyses of PAL activity

**Figure 6:**
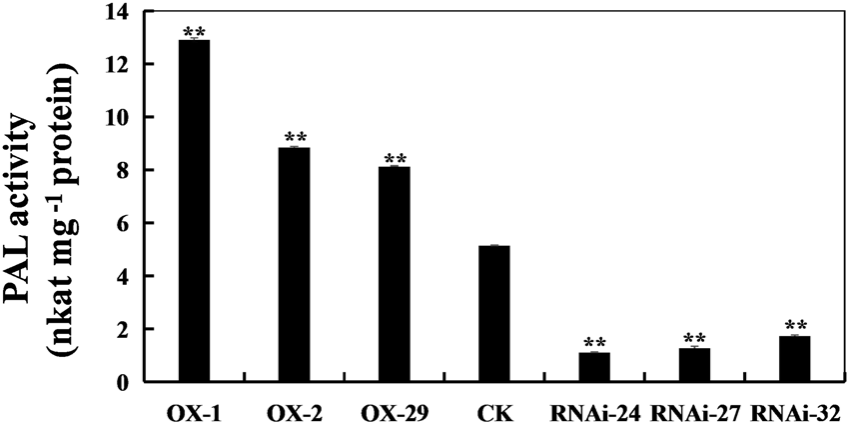
PAL activity in non-transgenic and transgenic soybean leaves treated with *P*. *sojae* inoculum at 36 h. The non-transgenic soybean plants were used as controls. The experiment was performed on three biological replicates with their respective three technical replicates and statistically analyzed using Student’s t-test (*P < 0.05, **P < 0.01). Bars indicate the standard error of the mean.

To determine whether there are changes in the PAL activity in *GmPAL2*.*1*-transgenic soybean leaves during *P*. *sojae* infection, the PAL activity was analyzed after 36 h of incubation with *P*. *sojae*. As shown in Fig. 6, the PAL activity in *GmPAL2*.*1*-OX transgenic soybean is significantly higher than that of non-transgenic plant leaves after infection with *P*. *sojae*. In all the *GmPAL2*.*1*-RNAi soybean lines, the PAL activity is distinctly compromised by *P*. *sojae* infection compared with that in non-transgenic plants. These results indicate that the expression of *GmPAL2*.*1* affects PAL activity in soybean leaves after infection with *P*. *sojae*.

### Isoflavone and glyceollin levels in transgenic soybean seeds

**Figure 7:**
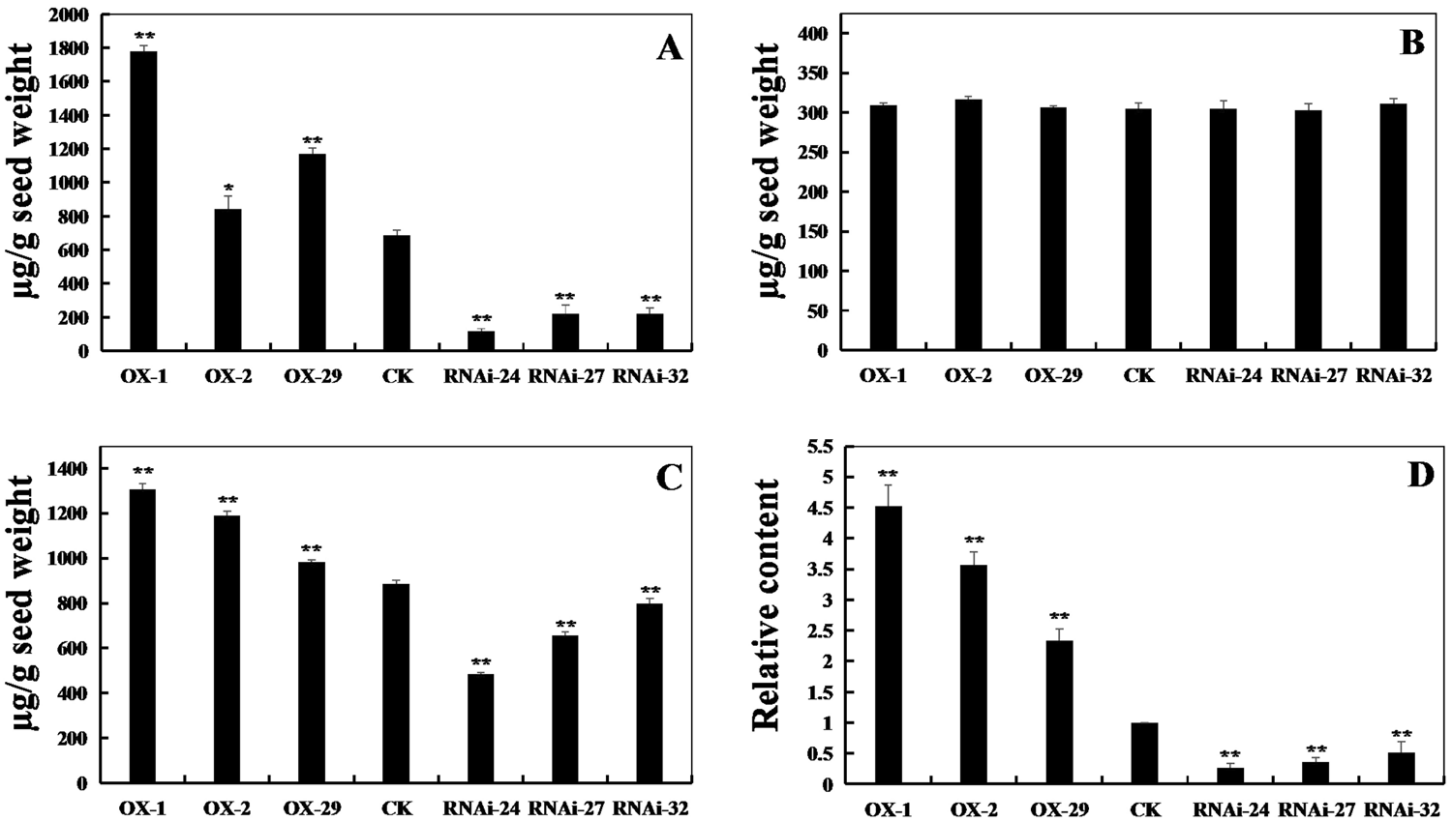
The content of the isoflavone components and the relative content of glyceollins in seeds of transgenic and non-transgenic soybeans. (**A**) The daidzein levels in seeds of transgenic and non-transgenic soybeans. (**B**) The glycitein levels in seeds of transgenic and non-transgenic soybeans. (**C**) The genistein levels in seeds of transgenic and non-transgenic soybeans. (**D**) The relative content of glyceollins in the seeds of transgenic and non-transgenic soybeans. Non-transgenic soybean plants were used as controls. The experiment was performed using three biological replicates with their respective three technical replicates and statistically analyzed using Student’s t-test (*P < 0.05, **P < 0.01). Bars indicate the standard error of the mean.

To test whether the *GmPAL2*.*1* expression level can cause change in the isoflavone and glyceollins content in soybean, the contents of three kinds of isoflavones (daidzein, genistein and glycitein) and the relative content of the glyceollins were measured in the seeds of transgenic soybean plants and non-transgenic soybean plants. The results show that the daidzein and genistein levels in the *GmPAL2*.*1*-overexpressing soybean plants are significantly higher than those of non-transgenic plants, and those levels are significantly compromised in *GmPAL2*.*1*-RNAi soybean plants (Fig. 7A,C). However, the levels of glycitein show little change compared to those of the control (Fig. 7B). As shown in Fig. 7D, the relative content of glyceollins in transgenic soybean seeds also varies markedly compared with that of the control. These results suggest that GmPAL2.1 may play a role in the defense resistance to *P*. *sojae* by participating in synthesis of isoflavones and glyceollins.

### The expression of SA marker genes and SA accumulation in transgenic soybeans

**Figure 8:**
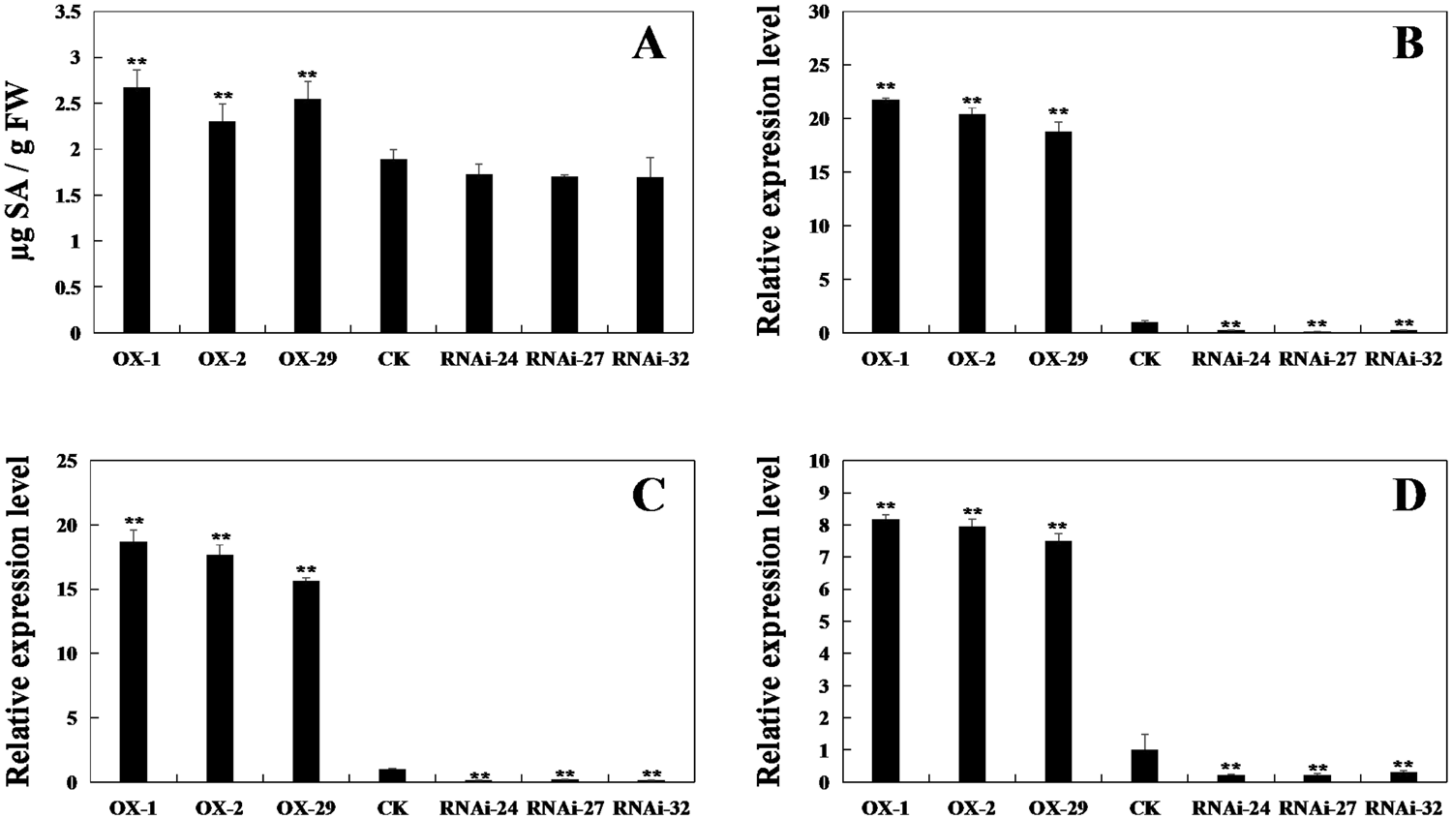
The content of SA and expression analysis of *GmNPR1*, *GmPR1* and *GmPR5* in transgenic and non-transgenic soybeans. The content of SA in leaves of transgenic and non-transgenic soybeans. (**B**) The relative transcript abundance of *GmNPR1* (NM_001251745) in transgenic and non-transgenic soybeans. (**C**) The relative transcript abundance of *GmPR1* (AF136636) in transgenic and non-transgenic soybeans. (**D**) The relative transcript abundance of *GmPR5* (M21297) in transgenic and non-transgenic soybeans. The amplification of the soybean *Actin* (*GmActin4*) gene was used as an internal control to normalize all the data. Three technical replicates were averaged and statistically analyzed using Student’s t-test (**P < 0.01). Bars indicate the standard error of the mean. The data are the means ± SD from three independent experiments. Asterisks indicate significant differences as determined by Student’s t-test (P < 0.05).

To test whether the GmPAL2.1 protein could regulate SA marker genes and SA accumulation, the content of SA and the expression of *GmNPR1*, *GmPR1*, *GmPR2* and *GmPR5* genes were analyzed. As shown in Fig. 8A, the SA accumulation in *GmPAL2*.*1*-OX transgenic soybean leaves is significantly higher than that of non-transgenic plants and *GmPAL2*.*1*-RNAi soybean plants. The transcripts of *GmNPR1*, *GmPR1 and GmPR5* changed significantly in all the transgenic lines compared with the control (Fig. 8B,C,D). Although there is difference in the content of SA between non-transgenic plants and *GmPAL2*.*1*-RNAi soybean plants, it does not reach a significant level.

## Discussion

In this study, we isolated and functionally characterized the PAL gene (*GmPAL2*.*1*), which acts as a positive regulator in resistance to *P*. *sojae* in soybean (*Glycine max*) plants. PAL, an entry-point enzyme in the phenylpropanoid biosynthesis pathway, was first isolated from barley (*Hordeum vulgare* L.)^53^. Since then, there have been many reports concerning the biochemical characterization and structures of PAL proteins in organisms, such as the PAL from *Petroselinum crispum*, *Arabidopsis thaliana*, *Streptomyces maritimus*, *Rhodobacter sphaeroides*, *Cyanobacteria*, *Rhodotorula glutinis*, and *Musa paradisiaca* ^26, 54–59^. In soybean, the gene encoding PAL was cloned by Frank and Vodkin in 1991^36^. However, there is little knowledge about the biological function of PAL in soybean. Here, we report for the first time that *GmPAL2*.*1* transgenic soybean plants inoculated with *P*. *sojae* display significantly altered responses to pathogen infection.

There are many studies that show *PAL* genes are involved in the response of plants to infection by pathogens^9, 60, 61^. In *Arabidopsis thaliana*, a pal1/pal2/pal3/pal4 quadruple knockout mutant showed increased susceptibility to the virulent bacterial pathogen *Pseudomonas syringae* ^40^. In transgenic tobacco, it has been reported that a partial suppression of the PAL gene gives rise to increased fungal susceptibility^55^. In the present study, we determined that the overexpression of *GmPAL2*.*1* transgenic soybean improves resistance to *P*. *sojae* and *GmPAL2*.*1*-RNAi soybean plants exhibits increased susceptibility. Moreover, PAL has been proposed to play important roles in biotic and abiotic stress responses in plants^3, 41, 43, 62^. In this work, the transcript abundance of *GmPAL2*.*1* following various stress treatments was analyzed. The results show that inoculation with *P*. *sojae* as a biotic stress and UV-B radiation, cold and dark treatments as abiotic stresses significantly increase the accumulation of *GmPAL2*.*1* mRNA in soybean plants (Fig. 1). This study also found that the transcript levels of *GmPAL2*.*1* are also remarkably increased by SA stress (Fig. 1), and further evidence showed that *GmPAL2*.*1*-transgenic soybean positively regulated the expression of the *GmNPR1*, *GmPR1* and *GmPR5* genes as well as SA accumulation (Fig. 8). *NPR1* has been identified to be involved in SA-mediated *PR* gene expression and resistance^63^. *PR1*, *PR2* and *PR5* are considered to be the effector genes for systemic acquired resistance (SAR), which was mediated by SA^64, 65^. It has been found recently that soybean sprouts germinated under red light improve resistance to *Pseudomonas putida 229* through the regulation of the de novo synthesis of SA and up-regulation of PR genes^66^. Therefore, we speculate that *GmPAL2*.*1* might play an important role in soybean plant resistance to *P*. *sojae*, depending mainly on the SA signaling pathway.

It has been reported that PAL is one of the branch point enzymes between primary and secondary metabolism^67^. The gateway from primary metabolism into phenylpropanoid metabolism is the deamination of L-phenylalanine by PAL to form trans-cinnamic acid^68^. The enzymatic activity of PAL determines the flux through the phenylpropanoid pathway and the rate of phenylpropanoid production, which has important functions in the plant defense against abiotic and biotic stresses^69–71^. Therefore, PAL activity has been suggested to play important roles in the plant defense against pathogens, typically as a physiological marker for measuring the resistance of plants, such as pea^72^, tomato^73^, cucumber^74^, and lupine^75, 76^. The determination of PAL activity also has great significance. A spectrophotometric assay was previously developed for testing the activity of PALs in plants, which accords to the formation of trans-cinnamic acid determined at 290 nm^77^. Some other similar spectrophotometric assays^78^ were also developed. In addition, studies have reported that the high performance liquid chromatography (HPLC) technique is a rapid and sensitive method to analyze PAL activity^79, 80^. In our study, the PAL activity in the leaf extract was determined by spectrophotometry following the method described by Song and Wang^81^. PAL activity in *GmPAL2*.*1*-OX soybean plants is significantly higher than that in non-transgenic plants after *P*. *sojae* infection and is markedly lower in *GmPAL2*.*1*-RNAi soybean plants (Fig. 6). Isoflavonoids belong to an important group of secondary metabolites derived from the phenylpropanoid pathway and play important roles in plant defense^82, 83^. To investigate whether the isoflavone content changes in the transgenic soybean lines, six T_2_ transgenic soybean seeds (OX-1, OX-2, OX-29, RNAi-24, RNAi-27 and RNAi-32) and non-transgenic soybean seeds were used to analyze the content of daidzein, glycitein and genistein. The results show that the daidzein content and the genistein content are positively and significantly correlated with the expression levels in the transgenic soybean seeds (Fig. 7A,C). But interestingly, it’s been found that the combination of transcription factor activation of multiple phenylpropanoid pathway genes and cosuppression of a single gene, F3H, the enzyme catalyzes the conversion of flavanones to dihydroflavonols, provided an effective metabolic engineering strategy for producing high levels of isoflavones in soybean seed^23^. IFR is an enzyme involved in the synthesis of glyceollins from daidzein and the daidzein content greatly reduced in *GmIFR*-overexpression soybean seeds^84^. The reason is maybe PAL produces trans-cinnamic acid, which serves as a precursor for the synthesis of all phenylpropanoids, including isoflavones^3^. These data suggest that the expression levels of *GmPAL2*.*1* might have an effect on PAL activity and the accumulation of isoflavones in response to *P*. *sojae* infection.

Several reports have shown that phytoalexins constitute a chemically heterogeneous group of low-molecular-weight antimicrobial compounds that are synthesized de novo and accumulate in plants in response to stress^85–88^. Glyceollins represent another group of phytoalexins whose biosynthesis is increased in soybean in response to various stress signals, such as fungal infection^87, 88^. Another study has suggested that the fungi *Aspergillus flavus*, *Aspergillus niger*, *Aspergillus oryzae*, and *Aspergillus flavus* are all capable of the inductive synthesis of glyceollins in soybean^89^. As isoflavonoid-type phytoalexins, glyceollins have exhibited antifungal activity^90^. Glyceollins have a significant antimicrobial effect against *Phytophthora capsici* and *Sclerotinia sclerotiorum* ^12, 14^ and exhibit resistance to *Phytophthora megasperma* var. *sojae* in soybean^91–93^. Previous research suggests that the biosynthesis of glyceollin is via the isoflavonoid branch of the phenylpropanoid pathway^94^. More specifically, the glyceollin biosynthetic pathway includes the enzymes involved in phenylpropanoid metabolism, flavonoid/isoflavonoid synthesis and those dedicated to the biosynthesis of pterocarpan phytoalexins^95^. Thus, PAL involves the synthesis of glyceollins, which are a mixture of structurally related pterocarpans^7, 96^. In this work, we detected the relative content of glyceollins in transgenic soybean seeds and nontransgenic soybean seeds. The relative content of glyceollins in *GmPAL2*.*1*-OX soybean plants is significantly higher than that in non-transgenic plants, while that in *GmPAL2*.*1*-RNAi soybean plants is lower (Fig. 7D). Therefore, we suggest that *GmPAL2*.*1* may play an important role in the biosynthesis of glyceollins to improve resistance to *P*. *sojae* in soybean.

## Methods

### Plant materials and stress treatments

The soybean cultivar ‘Suinong 10’, which is resistant to dominant physiological race 1 of *P*. *sojae* in Heilongjiang, China^97^, was used in this study. The seeds were grown in a glasshouse maintained at 22 °C and 70% relative humidity under a photoperiod of 16/8 h light/dark. Fourteen days after planting, seedlings at the first-node stage (V1)^98^ were used for various treatments.

For abiotic treatments, soybean leaves were each subjected to seven different stresses including UV radiation, low temperature (4 °C), dark treatment, ABA, SA, GA and MeJA. For UV treatment, the seedlings were exposed for 0, 3, 6, 9, 12, 24 or 36 h to an ultraviolet lamp. For low-temperature (4 °C) and dark treatments, soybean seedlings were incubated separately in a cold chamber and a darkroom for 0, 6, 12, 24, 36, 48 or 72 h. Untreated leaves of soybean were used as controls. ABA (50 µM), SA (0.2 mM), GA (250 mg L^−1^) and MeJA (100 µM) were dissolved in 0.01% Tween 20 and sprayed onto young leaves for 0, 1, 3, 6, 9, 12 or 24 h. The control leaves were sprayed with an equivalent volume of 0.01% (v/v) Tween 20. For *P*. *sojae* treatment, the soybean plants were infected with *P*. *sojae* race 1 using the procedure of Ward *et al*.^99^ and Morris *et al*.^100^ with minor modifications. Zoospores were developed following the method described by Ward *et al*.^99^, and the concentration was estimated using a hemocytometer to approximately 1 × 10^5^ spores mL^−1^. The unifoliolate leaves were harvested at 0, 1, 3, 6, 9, 12, 20, 24, 30, 36, 48 and 72 h after inoculation.

‘Dongnong 50’ soybean, which is susceptible to *P*. *sojae* dominant physiological race 1 and was provided by the Key Laboratory of Soybean Biology in the Chinese Ministry of Education, Harbin, was used for *P*. *sojae* treatment and gene transformation experiments.

### Isolation of the *GmPAL2*.*1* gene

A suppression subtractive hybridization library coupled with cDNA microarrays was queried using a soybean expressed sequence tag (EST) encoding an EST homologous to a phenylalanine ammonia-lyase from *Lotus japonicus*, previously shown to be upregulated in the highly resistant soybean ‘Suinong 10’ infected with *P*. *sojae* ^46^. Here, the full-length cDNA (termed *GmPAL2*.*1*, GenBank accession no. NM_001250027, NCBI protein no. NP_001236956) of the EST was amplified using RT-PCR with the cDNA of ‘Suinong 10’ using the primer pairs *GmPAL2*.*1F* and *GmPAL2*.*1R* (see Supplementary Table 1 for primer sequences). The primers for *GmPAL2*.*1* were used for PCR under the following condition: 94 °C for 5 min, followed by 30 cycles of 94 °C for 30 s, 68 °C for 30 s, and 72 °C for 30 s, with a final extension at 72 °C for 8 min. The amplification product was gel purified and cloned into the PMD18-T vector (TaKaRa, Dalian, China), then transformed into *E*. *coli* DH5α cells (Shanghai Biotech Inc, Shanghai, China) and sequenced (GENEWIZ, Beijing, China). Sequence alignments were performed using DNAMAN software (http://www.lynnon.com/). A phylogenetic analysis of *GmPAL2*.*1* and various heterologous PAL proteins was performed using MEGA4 software^101^. The three-dimensional (3D) structure of GmPAL was predicted using the online program Phyre2 (http://www.sbg.bio.ic.ac.uk/phyre2).

### Quantitative RT-PCR

For expression analysis of *GmPAL2*.*1* under abiotic and biotic stresses, the total RNA was isolated from ‘Suinong 10’ soybean leaves using TRlzol reagent ((Invitrogen, Shanghai, China). The first-strand cDNAs were synthesized using 1 µg of RNA with the Moloney murine leukemia virus reverse transcriptase kit (Takara, Dalian, China) according to the manufacturer’s protocol. The qRT-PCR analysis was performed using a real-time RT-PCR kit (Takara, Japan) with a CFX96 Touch^TM^ Real-Time PCR Detection System (BioBad, USA). DNA accumulation was measured using SYBR Green as the reference dye. The soybean housekeeping gene *GmActin4* (GenBank accession no. AF049106) was used as the internal control. Each qRT-PCR was run in three technical replicates.

### Subcellular localization

To investigate the subcellular localization of GmPAL2.1, the full-length *GmPAL2*.*1* was cloned in frame into the 5′-terminus of the GFP coding sequence in the 35 S:GFP vector using the primer pairs GmPAL2.1-GF and GmPAL2.1-GR (Supplementary Table 1), generating the fusion construct 35 S:GmPAL2.1-GFP. Arabidopsis protoplasts were acquired using the method described by Lin^102^. *Arabidopsis* protoplast transformation was performed as described by Yoo *et al*.^103^ with minor modifications. After incubation of the transfected Arabidopsis protoplasts cells for 16 h at 25 °C, the GFP signal was imaged using a TCS SP2 confocal spectral microscope imaging system (Leica, Germany).

### Vector construction and transformation of soybean

The full length *GmPAL2*.*1* coding region was amplified with two specific primers, *GmPAL2*.*1*-*F* and *GmPAL2*.*1*-*R* (Supplementary Table 1), for transformation assays. The following PCR cycling parameters were used: 94 °C for 3 min, 35 cycles of 94 °C for 30 s, 56 °C for 30 s, 72 °C for 1 min 30 s and a final cycle at 72 °C for 8 min. To overexpress the *GmPAL2*.*1* gene, the open reading frame of *GmPAL2*.*1* was cloned in frame into the pCAMBIA3301 vector. To suppress the gene expression of *GmPAL2*.*1*, *GmPAL2*.*1*-*attB*-*F* and *GmPAL2*.*1*-*attB*-*R* (Supplementary Table 1) were designed to amplify 310 bp fragments of *GmPAL2*.*1*. The fragments were cloned into the pjawoh18 vector. The plant expression vector was introduced into *Agrobacterium tumefaciens* LBA4404 and EHA105 using the freezing and thawing method as described by Holsters *et al*.^104^. ‘Dongnong 50’ soybean was used for the gene transformation experiments using the *Agrobacterium*-mediated transformation method described by Paz *et al*.^105^. To confirm transgene insertion in the soybean plants, genomic DNA was extracted from the transformants, and PCR analysis was conducted. Transgenic soybean plants (T_1_) were identified by PCR amplification and Southern blot hybrid-ization using a DIG High Prime DNA Labeling and Detection Starter kit II (Roche, Germany). Transgenic soybean plants (T_2_) were also identified by quantitative RT-PCR (see Supplementary Table 1 for primer sequences).

### Pathogen response assays of transgenic soybean plants

To investigate whether the *GmPAL2*.*1*-transformed plants have changes in resistance to pathogen infection, artificial inoculation procedures were performed according to the methods described by Dou *et al*.^106^ and Morrison and Thorne^107^ with minor modifications. The roots and living cotyledons of three T_2_ *GmPAL2*.*1*-overexpressing soybean plants (OX-1, OX-2, and OX-29) and three *GmPAL2*.*1*-RNAi soybean plants (RNAi-24, RNAi-27, and RNAi-32) were treated with a *P*. *sojae* inoculum. The roots and living cotyledons were incubated in a mist chamber at 25 °C with 90% relative humidity under a 14 h photoperiod at a light intensity of 350 µmol photons m^−1^ s^−1^ for investigation. The cotyledons of non-transformed plants were used as controls. Disease symptoms on each cotyledon were observed and photographed after inoculation using a Nikon D7000 camera.

To further determine the responses of *GmPAL2*.*1*-transformed soybean plants to *P*. *sojae* ingress, the relative biomass of *P*. *sojae* in infected cotyledons of the selected T_2_ transgenic plants at the first-node stage (V1)^98^ were assessed after 24 h, 48 h and 96 h of incubation with zoospore suspensions of *P*. *sojae*. The assessment of the biomass of *P*. *sojae* was based on the transcript level of *P*. *sojae TEF1* (GenBank accession no. EU079791) in reference to soybean *EF1β* according to the method of Chacón *et al*.^108^ (see Supplementary Table 1 for the *TEF1* and *EF1β* primer sequences). The pathogen response assays were performed on three biological replicates with their respective three technical replicates.

### PAL activity assay

Enzymes were extracted from four-week-old soybean seedlings leaves using 100 mM phosphate buffer (pH 6.0) containing 2 mM EDTA, 4 mM dithiothreitol, and 2% (w/w) polyvinylpyrrolidone. Fresh leaf samples were ground on ice for 5 min in 0.25 g · mL^−1^ of extraction buffer and then centrifuged for 25 min at 17,000 × *g* and 4 °C to obtain a solid-free extract. The PAL activity in the leaf extract was determined by the method of Song and Wang^81^, with slight modifications. Briefly, the protein extract (0.2 mL) was incubated at 30 °C for 60 min with 2 mL of 0.01 M borate buffer (pH 8.7) and 1 mL of 0.02 M L-phenylalanine (pre-dissolved in 0.01 M borate buffer, pH 8.7). This reaction was stopped by the addition of 1 mL of 6 M HCl. The reaction was then centrifuged for 10 min at 12,000× *g* to pellet the denatured protein. The absorbance was measured at 290 nm before and after incubation. One unit of activity (katal) was defined as the amount of PAL that produces 1 mole of cinnamic acid in 1 s and was expressed as nkat mg^−1^ of protein. A reaction without the substrate was our blank control. Triplicate assays were performed for each extract. The protein concentration was determined using the dye-binding Bradford method^109^ with bovine serum albumin as the protein standard.

### Isoflavone and glyceollin analysis

Approximately 0.1 g sample of seeds from T_2_ transgenic soybean plants (lines OX-1, OX-2, OX-29, RNAi-24, RNAi-27 and RNAi-32) was used to analyze the content of daidzein, glycitein and genistein. The three kinds of isoflavones were extracted from the samples and separated using HPLC as described by Zeng *et al*.^110^.

Seeds of T_2_ transgenic soybean plants (lines OX-1, OX-2, OX-29, RNAi-24, RNAi-27 and RNAi-32) were used for glyceollin extraction with 80% ethanol following the method described by Boue *et al*.^111^ and isolated using HPLC as described by Zeng *et al*.^110^. Non-transformed seeds extracts were used as controls.

### SA measurement

SA was extracted and measured from soybean plant leaves, as described previously by Aboul *et al*.^112^. Leaf tissues (0.5 g) were extracted in 1 mL of 90% methanol following homogenization in liquid nitrogen. 3-Hydroxybenzoic acid (Sigma) was used as an internal standard. The SA extracts were analyzed automatically using a fluorescence detector (excitation at 305 nm and emission at 405 nm) with reversed-phase high-performance liquid chromatography on a Waters 515 system (Waters, Milford, MA, USA) with a C18 column.

## Electronic supplementary material

Supplementary Information
