# Supplementary material for: Phenylalanine ammonia-lyase2.1 contributes to the soybean response towards Phytophthora sojae infection
Source: Sci Rep. 2017 Aug 3;7:7242. doi: 10.1038/s41598-017-07832-2 (PMC5543151; doi:10.1038/s41598-017-07832-2)
Supplement: Supplementary file 1 — Supplementary Information [file 41598_2017_7832_MOESM1_ESM.doc]

**Phenylalanine ammonia-lyase2.1 contributes to the soybean response towards *Phytophthora sojae* infection**

**Chuanzhong Zhang^1, †^, Xin Wang^1, 2†^, Feng Zhang^3†^, Lidong Dong^1^, Junjiang Wu^4^, Qun Cheng^1^, Dongyue Qi^1^, Xiaofei Yan^1^ , Liangyu Jiang^1^, Sujie Fan^1^, Ninghui Li^1, 5^, Dongmei Li^1^, Pengfei Xu^1, *^and Shuzhen Zhang^1, *^**

^1^Soybean Research Institute, Key Laboratory of Soybean Biology of Chinese Education Ministry, Northeast Agricultural University, Harbin, Heilongjiang, China

^2^Heilongjiang Academy of Land Reclamation Sciences, Harbin, Heilongjiang, China

^3^First Affiliated Hospital of Harbin Medical University, Harbin, Heilongjiang, China

^4^Soybean Research Institute of Heilongjiang Academy of Agricultural Sciences, Key Laboratory of Soybean Cultivation of Ministry of Agriculture P. R. China, Harbin Heilongjiang, China

^5^Jiamusi Branch Academy of Heilongjiang Academy of Agricultural Sciences, Jiamusi, Heilongjiang, China

**^†^**Chuanzhong Zhang, Xin Wang, Feng Zhang contributed equally to this research.

**^*^** Correspondence:

Shuzhen Zhang and Pengfei Xu, Soybean Research Institute, Key Laboratory of Soybean Biology of Chinese Education Ministry, Northeast Agricultural University, Harbin, Heilongjiang, China, 150030

E-mail: [zhangshuzhen@neau.edu.cn](mailto:zhangshuzhen@neau.edu.cn); [xupengfei@neau.edu.cn](mailto:xupengfei@neau.edu.cn)

**Supplementary** Table S1. Oligonucleotide primers used in this study.

| **Gene cloning** | *GmPALF* | GCTCTAGATCTCCCTCCACTCACCAT |
| --- | --- | --- |
|  | *GmPALR* | CGAGCTCGATTTGCCACAGCCTTAT |
|  | *GmPAL-attB-F* | GGGGACAAGTTTGTACAAAAAAGCAGGCTTTGGAAGGAAGCTCTTACGT |
|  | *GmPAL-attB-R* | GGGGACCACTTTGTACAAGAAAGCTGGGTTTACCAATTGAAGCAAGAGCC |
| **qRT-PCR** | *GmPAL-qF* | GCAGTGACTGGGTGATGG |
|  | *GmPAL-qR* | GCTCTGGTTGCTGTGTGG |
|  | *GmActin4F* | GTGTCAGCCATACTGTCCCCATTT |
|  | *GmActin4R* | GTTTCAAGCTCTTGCTCGTAATCA |
|  | *GmEF1F* | CCACTGCTGAAGAAGATGATGATG |
|  | *GmEF1R* | AAGGACAGAAGACTTGCCACTC |
|  | *GmTEF1F* | TGATCGTGCTGAACCACCC |
|  | *GmTEF1R* | CGAGCGACGGTCCATCTT |
|  | *GmNPR1F* | TCTTTGGGTTTTCGGTCT |
|  | *GmNPR1R* | CAACTTTCCTGCTTTCACA |
|  | *GmPR1F* | TGAAAATGTGGGTTGATGAGAAAT |
|  | *GmPR1R* | AAGTGATGAAAGTGCCTCCGTT |
|  | *GmPR5F* | CCCTCGCCTCCACTTCTTC |
|  | *GmPR5R* | TTGGTGCTCATCTTGCCTCTA |
| **GFP** | *GmPAL-GF* | GCAGATCTTCTCCCTCCACTCACCAT |
|  | *GmPAL-GR* | CACTAGTGATTTGCCACAGCCTTAT |

**Supplementary Figure S1. Nucleotide and amino acid sequences of *GmPAL2.1*.**


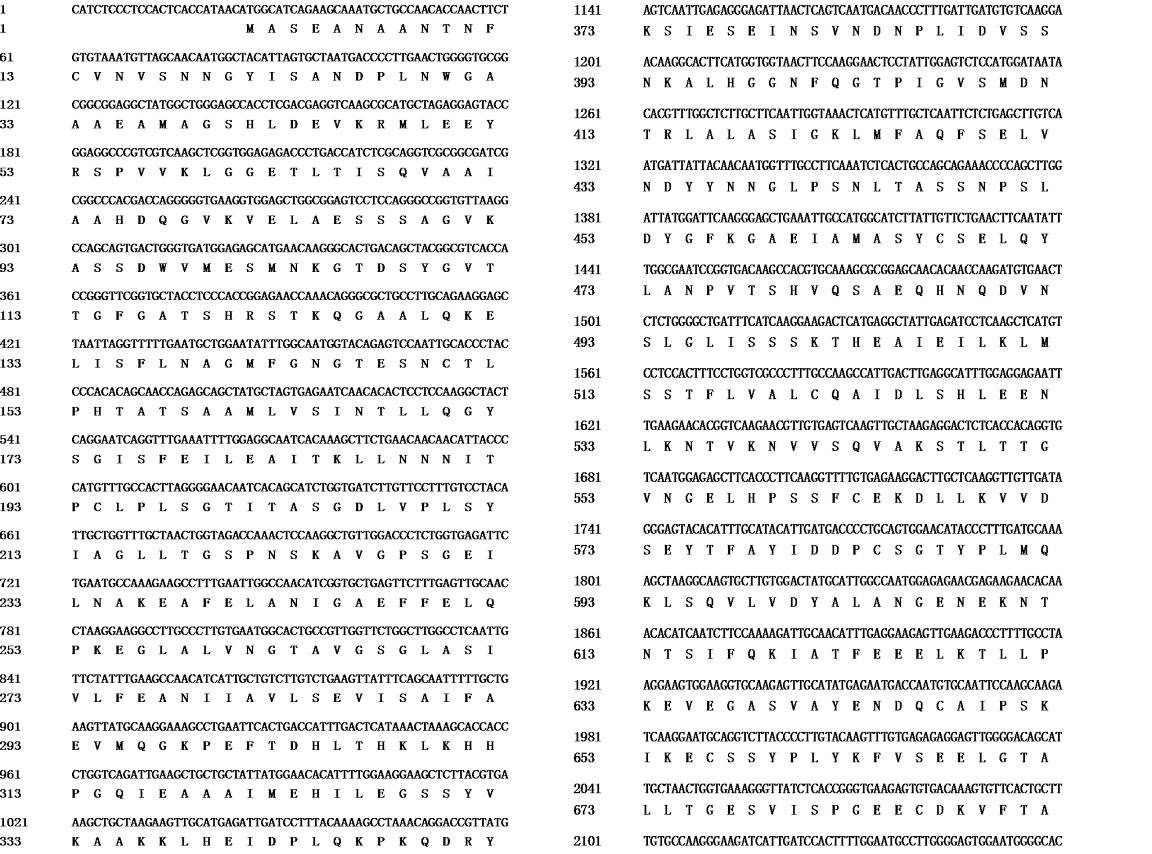


**Supplementary Figure S2. Cloning and characterization analysis of *GmPAL2.1*.** (A) Phylogenic analysis of the full-length amino acid sequence with other plant species. The GenBank accession numbers are as follows: LjPAL (BAF36972.1), AmPAL (EF567076.1), MtPAL (XM_003590423.1), TpPAL (AB236800.1), MePAL (AAK62030.1), DcPAL (BAG31931.1), FvPAL (XM_004304392.1), MaPAL (HM064433.1), RpPAL (ACF94716.1), PsPAL (D10001.1), OsPAL (NM_001054017.1), AtPAL (NM_111869.3), GmPAL1.1 (XM_003554334), GmPAL1.2 (XM_003521349), GmPAL1.3 (XM_003521348), GmPAL2.2 (XM_003556190), GmPAL2.3 (XM_003542493), GmPAL2.4 (XM_006589357), GmPAL3.1 (XM_003518532) (B) The predicted three-dimensional structure of the GmPAL2.1 protein. (C) Alignment analysis of the full-length amino acid sequence with other plant species.

**
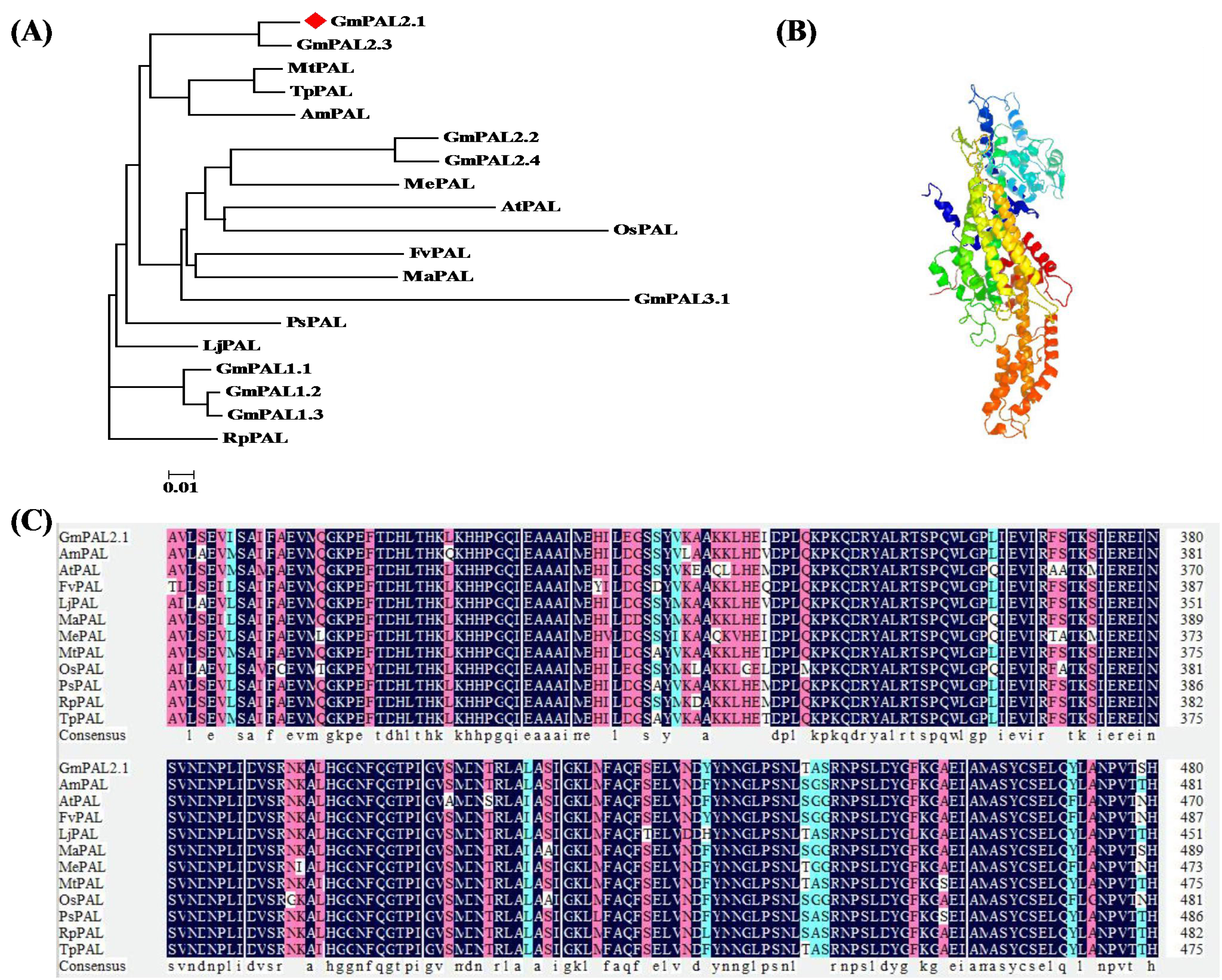
**

**Supplementary Figure S3. Alignment analysis of GmPAL2.1 and the homologs of *PAL2.1* gene in soybean.**

**
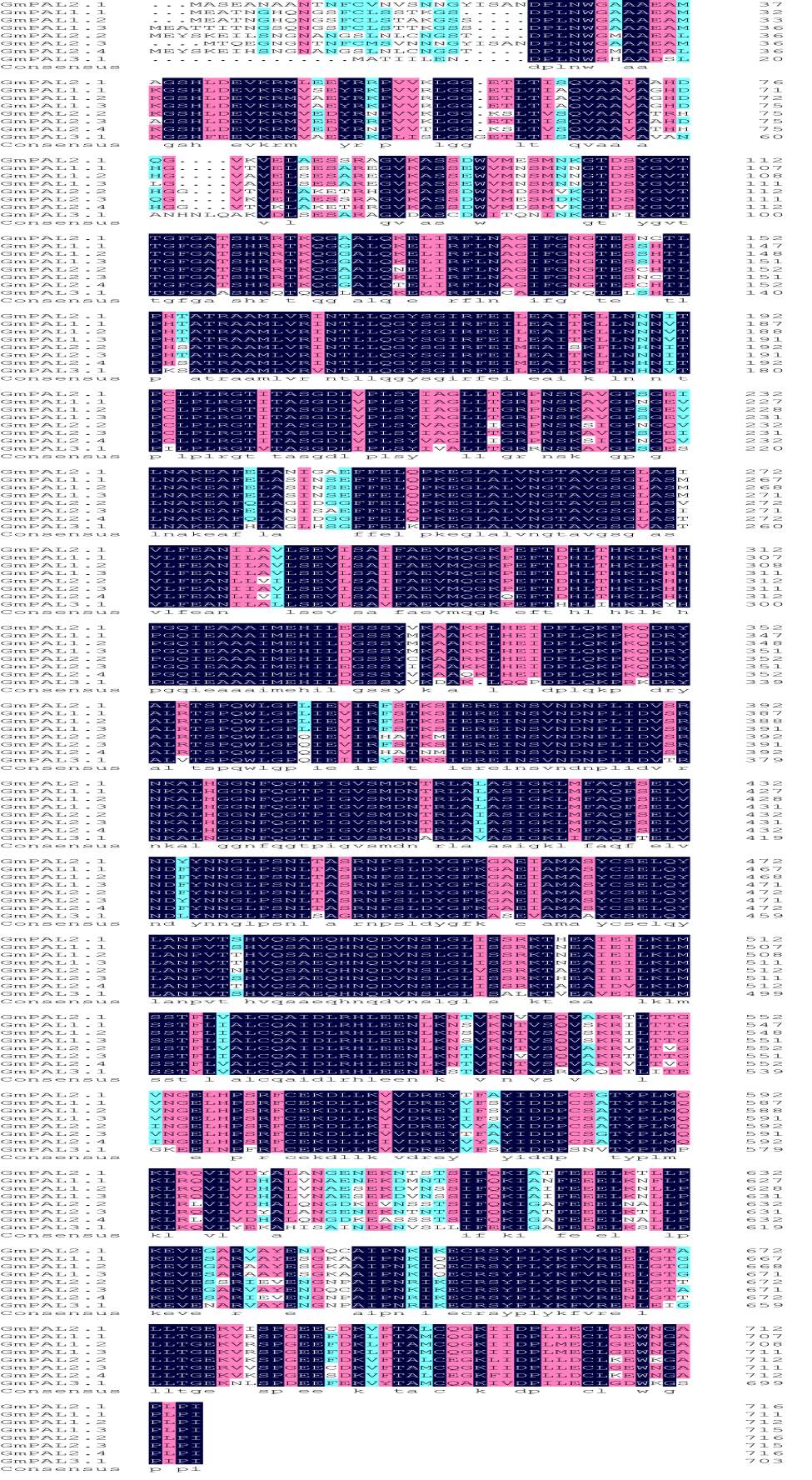
**

**Supplementary Figure S4. Southern blot assay of the T_1_ *GmPAL2.1*-transgenic soybeans.**

**
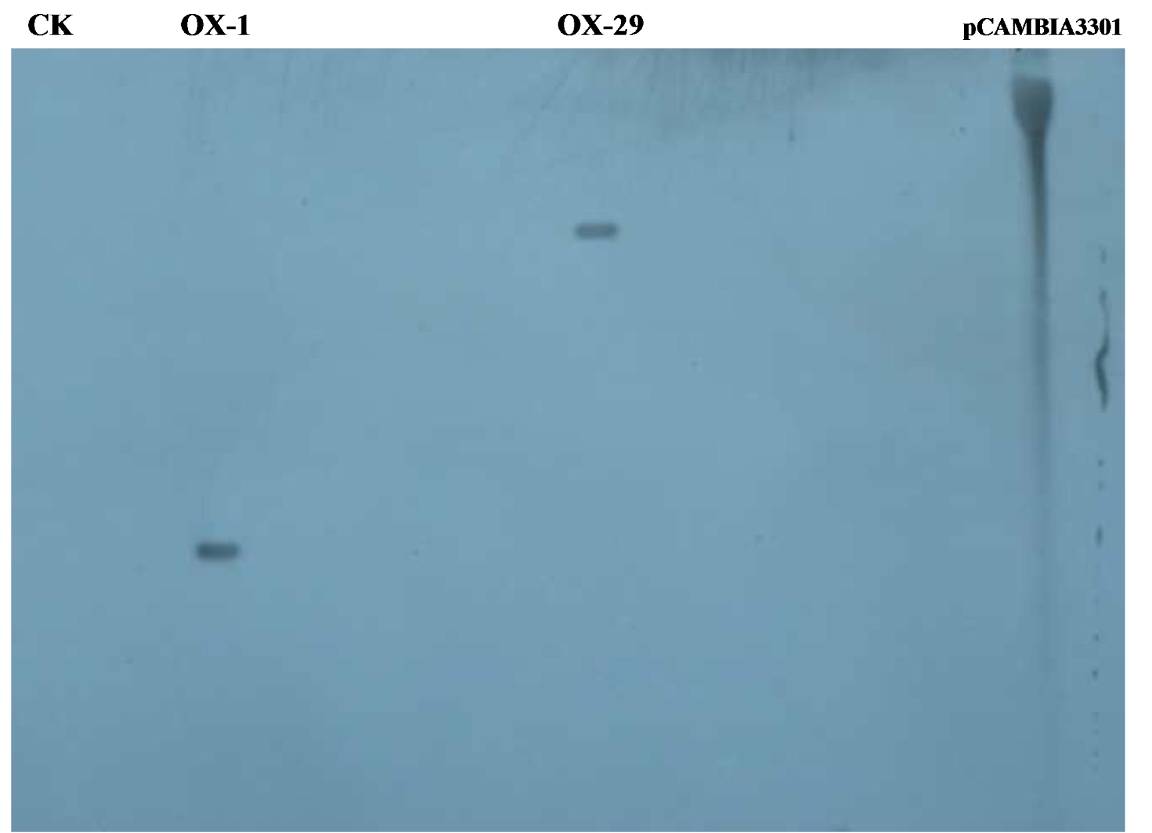
**
